# Effects of the surface irregularity of termite tunnels on food transport efficiency: a simulation study

**DOI:** 10.1093/jisesa/iead014

**Published:** 2023-03-22

**Authors:** Sang-Hee Lee, Cheol-Min Park, Sang-Bin Lee

**Affiliations:** Division of Industrial Mathematics, National Institute for Mathematical Sciences, Daejeon 34047, South Korea; Division of Industrial Mathematics, National Institute for Mathematical Sciences, Daejeon 34047, South Korea; Ft. Lauderdale Research and Education Center, Department of Entomology and Nematology, University of Florida, Ft. Lauderdale, FL 33314, USA

**Keywords:** termite, food transport efficiency, tunnel irregularity, traffic jam, individual-based model

## Abstract

Termites are believed to have evolved in a way that optimizes their foraging efficiency, which involves both searching for food and transporting it efficiently. Although the search efficiency has been well-studied through tunnel pattern analysis, transport efficiency has received limited attention due to the challenges of directly observing behavior that is highly influenced by environmental conditions. In this study, we introduce an individual-based model to simulate transport behavior and examine transport efficiency (*E*) by considering the tunnel surface irregularities and curvature, which are critical environmental factors. The model is characterized by four control variables: tunnel curvature (*k*_1_), termite stopping time at irregularity sites (*k*_2_), irregularity distribution (*k*_3_), and irregularity density (*k*_4_). The simulation results indicate that as *k*_1_ increases, *E* decreases, while *k*_3_ has little impact on *E*. The impact of *k*_4_ on *E* is decisive; when *k*_4_ ≤ 6, an increase in *k*_4_ results in increased traffic jam frequency and a faster reduction in *E*. However, when *k*_4_ > 6, the jamming frequency is not significantly affected, reducing the decrease in *E*. *k*_2_ strongly contributes to reducing *E* without significantly affecting the frequency. In the discussion section, we explore potential mechanisms that termites use to maintain transport efficiency in heterogeneous soils, and discuss how to improve the model to better reflect real-termite systems.

## Graphical Abstract

**Graphical Abstract:**
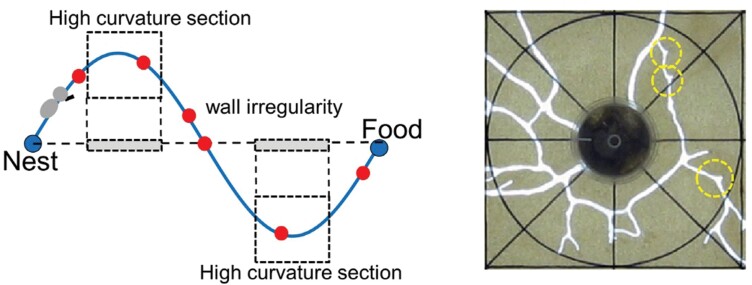

## Introduction

As one of the social insects, termites are believed to have evolved in a way that optimizes the strategic allocation of time and energy required for foraging (Campora and Grace 2001, Tschinkel 2011). Since foraging consists of food search efficiency and food transport efficiency, researchers have explored the termite system by examining these efficiencies (Campora and Grace 2004).

Some subterranean termites, such as *Coptotermes*, often construct underground tunnels over 100 meters in length to obtain food resources (King and Spink 1969, Su and Scheffrahn 1988). Therefore, if the two efficiencies are not optimized, the total energy they can use for foraging is greatly wasted, which is likely to be a threat to the survival of the species.

Of the two efficiencies, the search efficiency has received much more attention due to the practical need for termite control through tunnel pattern investigation (Hedlund and Henderson 1999, Puche and Su 2001). This investigation, conducted both experimentally and theoretically, showed that termites construct tunnels towards maximizing the food-finding probability, which reflects their search efficiency (Campora and Grace 2001). On the other hand, there have been few studies on transport efficiency so far because it is technically difficult to directly observe the food transport behavior affected by environmental conditions (Du et al. 2017, Su et al. 2017). Puche and Su (2001) briefly discussed transport efficiency through tunnel pattern analysis rather than through behavioral observation, and showed that the tunnel pattern has a fractal structure. It is well known that fractal structures are highly efficient for material flow and resource transport (Coleman and Vassilicos 2008, Sun et al. 2018), which indirectly suggests that termite tunnels have a high level of transport efficiency. However, the study was limited in that it could not provide any information on the relationship between environmental conditions (e.g., tunnel curvature and tunnel surface irregularities) and efficiency.

In this study, as an alternative to overcome the observational difficulties of food transport behavior at the individual level, we constructed a simulation model to mimic the transport process. This model specifically implemented individual-individual and individual-tunnel interactions. The individual-tunnel interaction included three control variables. Ku et al. (2012) found that when termites encountered irregularities on the tunnel surface while passing through the tunnel, they spent time touching the irregularities with their antennae to obtain structural information about the irregularities. Considering the length of the termite tunnel, the accumulated time would be quite long, which is likely to affect transport efficiency degradation. Thus, we chose irregularity density, irregularity distribution, and the spent time as control variables. Sim and Lee (2013) found that the more curved and wider the tunnel, the longer it took for termites to pass through. Therefore, it was inferred that tunnel curvature could significantly affect transport efficiency. For this reason, we selected tunnel curvature as an additional control variable. The individual-individual interaction was characterized by two behavioral processes in simulated termites. Anderson et al. (2002) theoretically showed that food transfer behavior between individuals contributes to increase transport efficiency under the condition that the distance between nest and food site is long. The transfer process is referred to as the bucket brigade mode. The mode was observed when subterranean termites carried excavated soil particles during tunneling (Mizumoto et al. 2020). Given that termites transport food over distances of several tens of meters or more (King and Spink 1969, Su and Scheffrahn 1988), termites are more likely to use the bucket brigade mode when transporting their food. Therefore, we designed the simulated termites to use this mode when transporting food.

Another behavioral process is traffic jamming caused by multiple termites moving in opposite directions within the tunnel. To the best of our knowledge, quantitative studies on termite traffic jams have not been carried out. Nevertheless, it is a reasonable inference that a high jamming frequency is likely to result in reduced transport efficiency, which is why we included the jamming process in the model.

In the discussion section, we briefly present the direction of experimental studies on termite food transport and discuss potential strategies that termites could take to optimize foraging efficiency. We also discuss how to make the model more realistic.

## Model Description and Analysis

### Individual-based Model Description

We made a simulation model to mimic the food transport behavior of termites at an individual level. The model consisted of a sinusoidal-shaped tunnel in a two-dimensional grid space with simulated termites transporting food along the tunnel. The use of pheromones was excluded for interactions between simulated termites. Real termites sometimes recognize the irregularity of the tunnel surface as a cue to excavate a new tunnel (Lee et al. 2008). However, in the model, we assumed that the tunnel reached a stationary state where the tunnel pattern does not change over time.

In the present study, we repeated the simulations 10 times for the combinatorial case for the variable values, and the simulation time *T* was set to 5,000. Additionally, the analysis results were averaged over the repetitions.

### Simulated Termite Response to Tunnel Irregularities

**Fig. 1.**
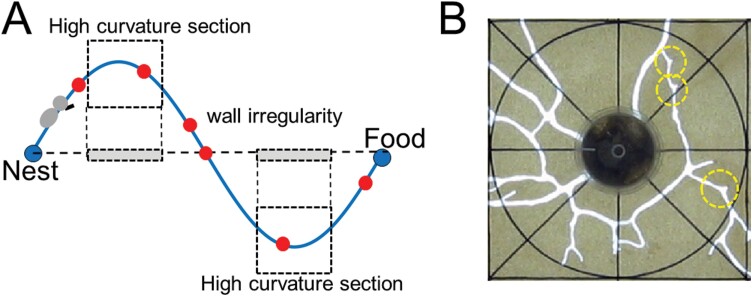
(A) Illustration showing a sinusoidal tunnel with a nest and food site at either end. Dotted rectangles indicate high curvature sections and red circles indicate tunnel surface irregularities. (B) Two transparent acrylic plates filled with sand (light brown) and a tunnel excavated by termites (white). The central black circle and yellow dotted circles are the termite introduction hall and tunnel irregularities, respectively.

We built a simulated tunnel that follows the shape of the function *y* = 20*sin* (*πx*/ 500) (*x* = 0, 1, …, 1,000) in grid cell space. The tunnel contained two sections with high curvature, which were set as regions 235 ≤ *x* ≤ 265 and 735 ≤ *x* ≤ 765 as shown in the dotted rectangle in Fig. 1.

Actual termites are known to slow their walking speed when passing through sections with high tunnel curvature (Sim and Lee 2013). To reflect the tunnel curvature effect in the model, we generated a random number between 0 and 1 each time the simulated termite walked within the section. The simulated termites were allowed to walk (stop) if the random number was greater (less) than the value of (*k*_1_ − 1) ×0.05. Therefore, as the value of *k*_1_ increased, the stop frequency increased. In this respect, *k*_1_ became the control variable that characterized the tunnel curvature effect. Here *k*_1_ = 1, 2, 3... 11.

Ku et al. (2012) experimentally found that when termites encounter irregularities on the tunnel surface, they stop walking for a few seconds. The authors also showed that the stopping time was correlated with the irregularity size. In this study, we assumed that the simulated tunnels had irregularities of various sizes. To implement this assumption in the model, we determined the stopping time using a Poisson probability density function.

This means that each stopping event is completely independent of the other stopping events, and the events occur at a constant average rate (Barbour and Hall 1984). The density function is written as:


$$\begin{array}{l} P(\tau )=\frac{exp\left(-\lambda \right)\times \lambda^{\tau }}{\tau !} \end{array}$$ (1)

Here *τ* and *λ* represent the stopping time and the average time for the stopping events, respectively. Since the value of *τ* is determined by the value of *λ*, the stopping behavior can be characterized by *λ*. In this model, we set the value of *λ* to be (*k*_2_ − 1) ×5, where *k*_2_ = 1, 2, 3... 11.

**Fig. 2.**
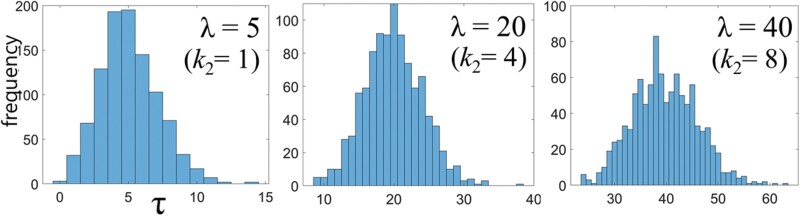
Poisson probability distribution function for the stopping time (*τ*) of simulated termites encountering an irregularity site. Here *k*_2_ (= 1, 4, and 8) represents the mean value of the distribution (*λ*).

Figure 2 shows the Poisson distribution function for the case of *k*_2_ = 1, 4, and 8. For *k*_2_ = 1 (4 or 8), the stopping frequency with *τ* values far from *λ* was relatively low (high).

Since there are still few studies on how the irregularities are formed on the tunnel surface, we confirmed through a simple experiment that irregularities with various sizes can be formed by termite tunneling behavior. We set two square transparent acrylic plates (with a gap of 2 mm) and filled the gap with homogeneous sand. The plates had a side length of 100 cm. We then introduced 10,000 termites (*Coptotermes formosanus* sp.) through a circular hole located in the middle of the plates.

The termites built a tunnel network for 24 h and reached a stationary state where the tunnels no longer changed over time. Figure 1B shows a square area with a length of 20 cm centered on the hole. In the figure, the black and white lines represent the guideline to visually show the degree of curvature of the tunnel and the tunnel excavated by the termites, respectively. The yellow dotted circle represents the tunnel irregularity. Termites sometimes stop tunneling behavior abruptly and move to another tunnel tip to start a new tunneling behavior. Further studies to understand the behavior of digging the new tunnel are beyond the scope of this study, thus we did not proceed further.

To distribute the irregularities on the simulated tunnel, we used the below function:


$$\begin{array}{l} y=sin\left(\frac{2\pi f}{L}x\right) \end{array}$$ (2)

**Fig. 3.**
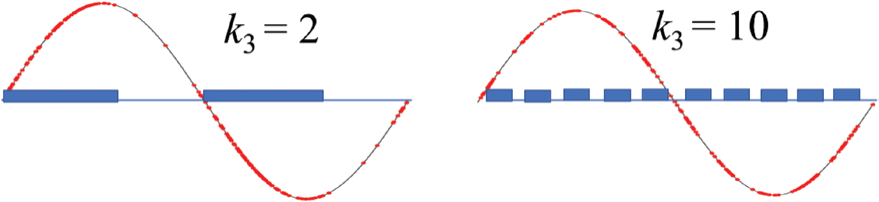
The location of the irregularities (red dots) on the simulated tunnel for *k*_3_ = 2 and 10. Areas with dense dots are indicated by bands on the *x*-axis.

*f* denotes the number of cycles of the function and *L* (= 1,000) is the tunnel length along the *x*-axis. We set *f* to have a value of (*k*_3_ – 1) × 2. For the regions where *y* > 0, we randomly distributed irregularities at (*k*_4_ – 1) × 30. Therefore, *k*_3_ and *k*_4_ represent the irregularity distribution and density, respectively. Here, *k*_3_ and *k*_4_ have values of 1, 2, 3… 11. Figure 3 shows the irregularity distribution for the cases, *k*_3_ = 2 and 10, where *k*_4_ = 8. To make it easier to visually see the area with high irregularity density, it is marked with a blue band on the *x*-axis.

We designed one end of the simulated tunnel to be connected to a food site with sufficient food for the duration of the simulation, and the other end to be connected to a nest site. Simulated termites can pick up only one food particle at a time and they were randomly placed in the tunnel at *t* = 0. One hundred simulated termites are initially introduced.

### Food Transport Behavior of Simulated Termites

**Fig. 4.**
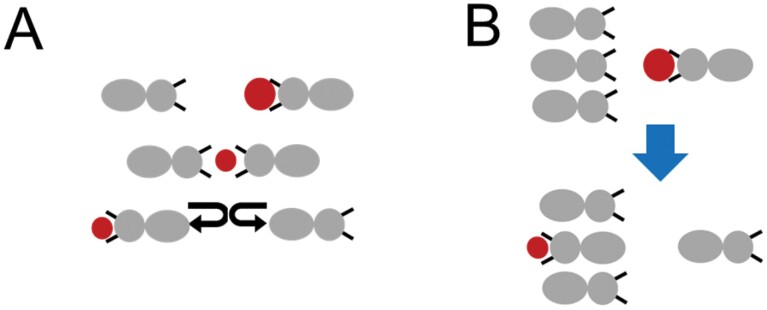
Illustration of a simulated termite transferring food to (A) one neighbor and (B) three neighbors. The large and small circles represent foods that did and did not suffer loss, respectively.

We allowed a food transfer between two termites to occur with a probability of 0.2 when a simulated termite with food met a termite without (Fig. 4). In this process, food loss is accompanied, and each loss decreases by 0.09. One food particle that did not suffer food loss had a value of 1.0. Through a preliminary study, we confirmed that the effect of the bucket brigade mode was negligible when the transfer probability was less than 0.1 and that food was hardly transported to the nest when the probability was greater than 0.5. For this reason, we appropriately selected the transfer probability value of 0.2.

Figure 4 shows the transfer process. The large and small red circles represent the food that did not suffer loss and the food that suffered loss, respectively. As soon as the transfer occurred, the direction of the two termites reversed. If an individual with food encountered two or more individuals without food at the same time, one of the individuals without food was randomly selected for food transfer (Fig. 4B). On the other hand, when two individuals with food met each other, no food transfer occurred. If food loss occurred *n* times during food transport, the food dropped into the nest was 1 – (*n*×0.09). When this value became negative, we considered it to be zero. The transport efficiency *E* was defined as follows:


$$\begin{array}{l} E\left(\tau \right)=\left(\frac{s\times \overset{\tau }{\underset{t=1}{\sum }}food\left(t\right)}{\tau \times N_{0}}\right) \end{array}$$ (3)

Here, ‘*food*(*t*)’ represents the amount of food particles accumulated in the nest by the simulated termites at time *t*, and *N*_0_ represents the number of termites participating in the food transport process. In addition, *s* is a constant having a value of 10,000 introduced to properly scale up *E* having a too-small value.

### Traffic Jam

To observe how a traffic jam occurs when termites pass through a narrow tunnel, we cut a straight artificial tunnel with a width of 2 mm and a length of 10 cm on a black acrylic plate and covered the top and bottom with two transparent acrylic plates. The gap between the top and bottom plate was 2 mm. Both ends of the tunnel were connected to circular halls with a diameter of 1 cm, and 10 termites were introduced through the hall.

**Fig. 5.**
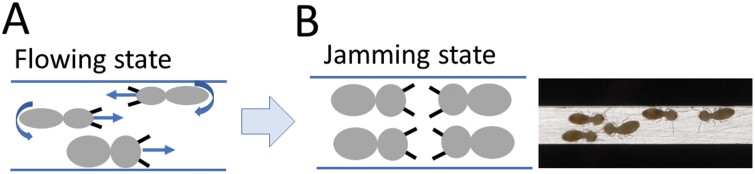
Illustration showing the conditions for simulated termites changing from a flow state to a traffic jamming state in a tunnel. The image on the right shows a real termite traffic jam.

When two termites encountered each other while walking in opposite directions, they passed without stopping by touching their antenna with each other. On the other hand, when 3 or more individuals met, they passed by turning their bodies vertically (Fig. 5A). The turning served to reduce the cross-sectional area of the tunnel width occupied by the individual. In cases where the advancing individuals had difficulty passing each other even with the turn, they were stuck facing each other for a few seconds to several minutes (Fig. 5B). In this study, we call this the traffic jam. To our knowledge, there are no quantitative studies on termite traffic jams so far. Nevertheless, we can infer that traffic jams are likely to affect transport efficiency. To implement a traffic jam in the model, we set up a simulation rule that a traffic jam occurs when four simulated termites meet at the same site and the sum of their direction vectors becomes zero. The individuals trapped in a traffic jam were then stopped for 100 time steps.

### Sensitivity Analysis

To investigate the size of the effect of four variables (*k*_1_, *k*_2,_*k*_3_, and *k*_4_) on food transport efficiency (*E*), the partial rank correlation coefficient (PRCC) method, one of the sensitivity analyses, was used as a sampling-based method (Marino et al. 2008). To obtain the PRCC value, we first need to calculate the correlation coefficient (CC) of the output *y* for the input *x*_j_ defined by the following formula:


$$\begin{array}{l} CC\left(x_{j},y\right)=\;\;\;\frac{\overset{N}{\underset{i=1}{\sum }}\left(x_{ij}-\bar{x}\right)\left(y_{i}-\bar{y}\right)}{\sqrt{\overset{N}{\underset{i=1}{\sum }}{\left(x_{ij}-\bar{x}\right)}^{2}\overset{N}{\underset{1}{\sum }}{\left(y_{i}-\bar{y}\right)}^{2}}},j=1,2,\ldots ,k \end{array}$$ (4)

Here, *N* and *k* represent the number of samples and the size of the input vector, respectively. *x* represents one or another of the four parameters *k*_1_, *k*_2_, *k*_3_, or *k*_4_, and *y* refers to the efficiency, *E*. The partial correlation coefficient (PCC), which eliminates the linear influence of other variables, measures the degree of relatedness between the input and the output. Therefore, PCC can be calculated as the CC of $x_{j}-{\hat{x}}_{j}\;$and *y*$-\hat{y}$. Here, ${\hat{x}}_{j}$and $\hat{y}\;$can be obtained using the following linear regression model


$$\begin{array}{l} {\hat{x}}_{j}=c_{0}+\underset{\begin{array}{l} p=1\\ p\neq j \end{array}}{\overset{N}{\sum }}\;c_{p}x_{p}\;and\;\hat{y}=b_{0}+\underset{\begin{array}{l} p=1\\ p\neq j \end{array}}{\overset{N}{\sum }}\;b_{p}x_{p}\; \end{array}$$ (5)

where *b* (and *c*) and ‘hat’ represent the regression coefficients and the regression fit variable, respectively. PRCC, which calculates the partial correlation using Eq. (5), has a value between –1 and 1. A positive (negative) value indicates a positive (negative) correlation between the parameter and the model output. On the other hand, the absolute PRCC value of each parameter indicates the degree of influence the parameter has on the output.

In this study, we used the LHS (Latin Hypercube Sampling) method, which has the advantage of avoiding repetitive sampling and significantly reducing the number of simulations (Iman and Helton 1988, McKay et al. 2000). We performed a total of 146,410 simulations for each of the combinations of values for the variables *k*_1_, *k*_2_, *k*_3_, and *k*_4_, repeating them 10 times each. For the calculation of Eqs. (4) and (5), the ‘prcc’ function provided in the 2021b version of MATLAB was used.

## Results

**Fig. 6.**
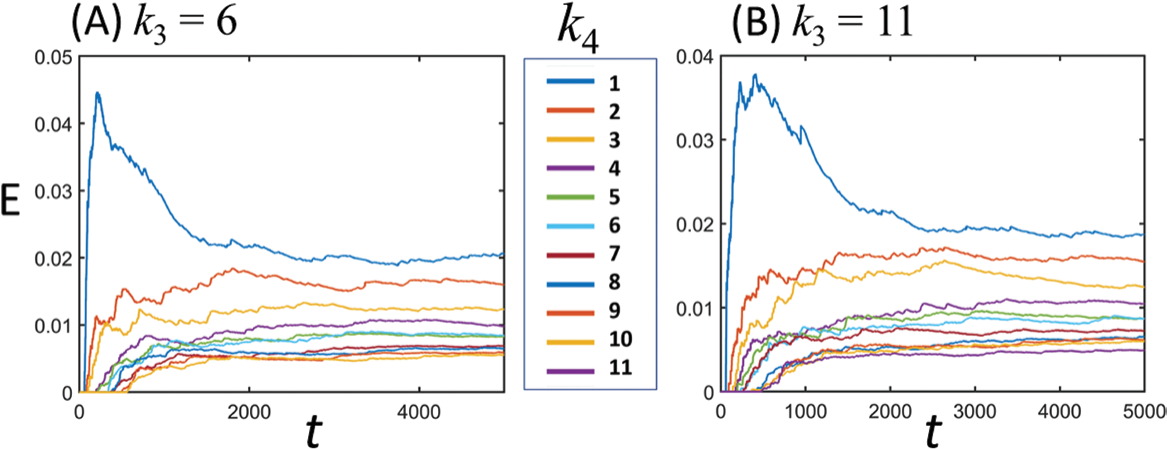
Changes in food transport efficiency, *E*, for the irregularity distribution (A) *k*_3_ = 6 and (B) *k*_3_ = 11. Here, the irregularity density, *k*_4_, has values of 1, 2, 3... 11.

**Fig. 7.**
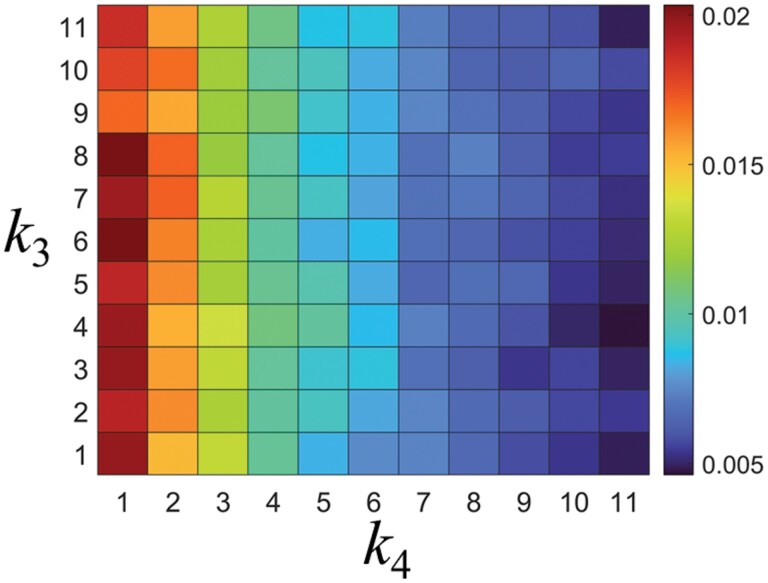
Food transport efficiencies, *E*, for tunnel irregularity distribution, *k*_3_, and irregularity density, *k*_4_.

Figure 6 shows the food transport efficiency*, E*, over time for both cases *k*_3_ = 6 and 11. The *E* value showed a tendency to become saturated in the time domain of *t* > 3,000 regardless of the *k*_4_ value. The saturated value decreased as *k*_4_ increased (Fig. 6A). When *k*_4_ = 1, the *E* rapidly increased to 0.045 and then gradually decreased. This is because the simulated termites were randomly placed throughout the tunnel at the beginning of the simulation (*t* = 0). The simulated termites closer to the food site were able to pick up the food more quickly than the individuals farther away. In the presence of irregularities (*k*_4_ > 1), the *E* value gradually increased in the range of *t* < 2,000 and then became saturated. This is because the simulated termites stopped walking at the irregularity sites, which diluted the initial distribution effect. Even in the case of *k*_3_ = 11, the *E* value showed a similar trend to that in the case of *k*_3_ = 6 (Fig. 6B). This indirectly shows that *k*_3_ has little effect on *E*, whereas *k*_4_ has a relatively greater effect on *E* than *k*_3_. We investigated *E*-maps for *k*_3_ (= 1, 2, 3… 11) and *k*_4_ (= 1, 2, 3... 11) to quantitatively compare the effects of the variables on *E* (Fig. 7). In the *E*-map, the *E* value decreased sharply as *k*_4_ increased but showed little change as *k*_3_ increased. This indicates that *k*_4_ is the variable that significantly contributes to *E*.

**Fig. 8.**
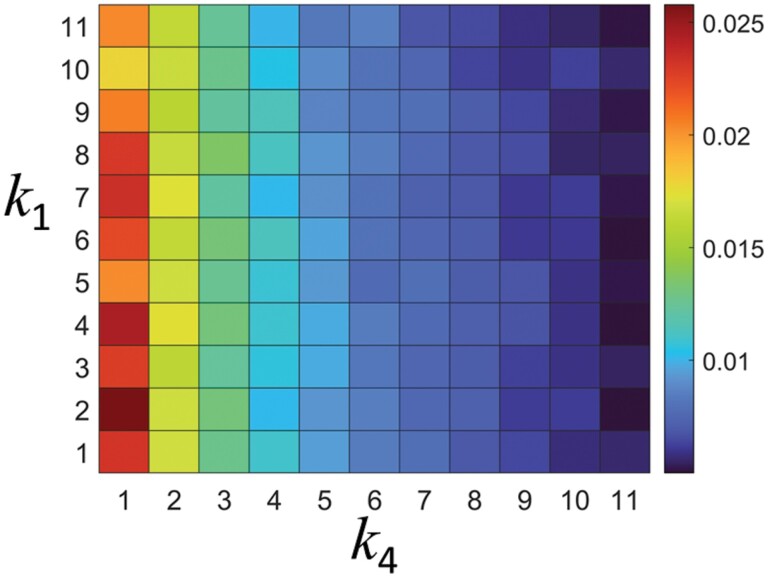
Food transport efficiencies, *E*, for the tunnel curvature effect, *k*_1_, and irregularity density, *k*_4_.

**Fig. 9.**
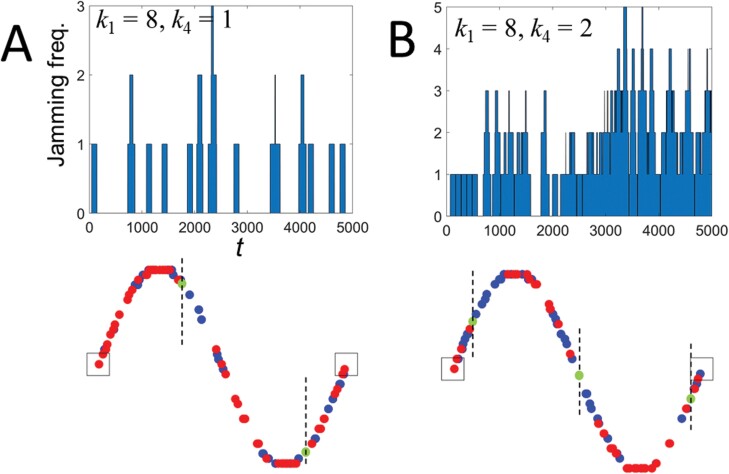
Traffic jam frequency (top) and jamming location (bottom) of simulated termites for tunnel irregularity densities (A) *k*_4_ = 1 and (B) *k*_4_ = 2. Red and blue circles represent individuals carrying food to the nest and individuals heading to the food site to pick up food, respectively. Green circles represent individuals in traffic jams.

Figure 8 shows an *E*-map for *k*_1_ and *k*_4_. *E* showed a tendency to decrease rapidly as *k*_4_ increased regardless of *k*_1_. This means that *k*_4_ has a significant effect on *E*. When *k*_4_ = 1, *E* clearly decreased as *k*_1_ increased. This is because the simulated termites frequently stopped walking in the high curvature section. However, for tunnels with irregularity (*k*_4_ > 1), simulated termites stopped walking not only by the tunnel curvature but also by the irregularity, which diluted the *k*_1_ effect. Traffic jams of simulated termites also contributed significantly to the sharp decrease between *k*_4_ = 1 and 2. To confirm this visually, we investigated the traffic jam frequency for the tunnels without irregularities (Fig. 9A) and those with irregularities (Fig. 9B). In the upper figure, the jamming frequency was much higher in the tunnel with irregularity (*k*_4_ = 2) than in the tunnel without irregularity (*k*_4_ = 1). The lower figure shows where the traffic jams occurred at specific times for *k*_1_ = 8, *k*_4_ = 1 (left) and *k*_1_ = 8, *k*_4_ = 2 (right). The red and blue circles represent individuals transporting the food to the nest and those heading to the food site to pick up food, respectively. The green circles represent individuals trapped in traffic jams. As mentioned in the model section, one green circle contains 4 simulated termites. Therefore, we can infer that the contribution of the traffic jam to the reduction of *E* is greater than that of the high curvature. In the tunnels without irregularities, the green circles appeared mainly in sections with high curvature, whereas in the tunnels with irregularities, the green circles tended to appear throughout the tunnel (see dotted straight lines).

**Fig. 10.**
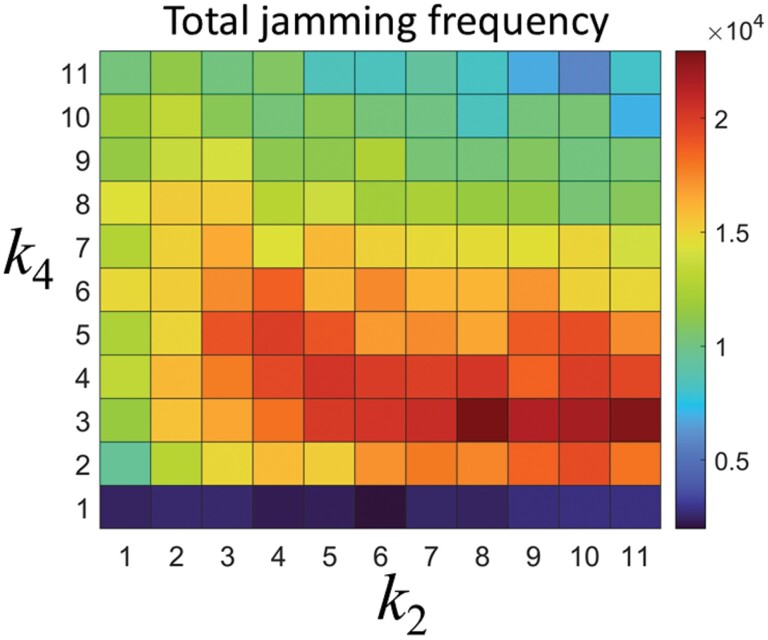
Total jamming frequency of the simulated termites for *k*_4_ and *k*_2_.

To examine the results of Fig. 9 more clearly, we investigated the total jamming frequency defined as the summation of the jamming frequency at each time step for *k*_4_ and *k*_2_ (Fig. 10). Interestingly, for *k*_4_ ≤ 6, the increase in the irregularity density increased the stopping frequency of simulated termites, which in turn increased the number of individual encounters. The encounters led to the occurrence of traffic jams. On the other hand, for *k*_4_ > 6, too high of an irregularity density stopped most individuals, reducing the number of encounters. Therefore, the total jamming frequency decreased while the total jamming frequency showed a tendency to slightly increase as the *k*_2_ value increased.

**Fig. 11.**
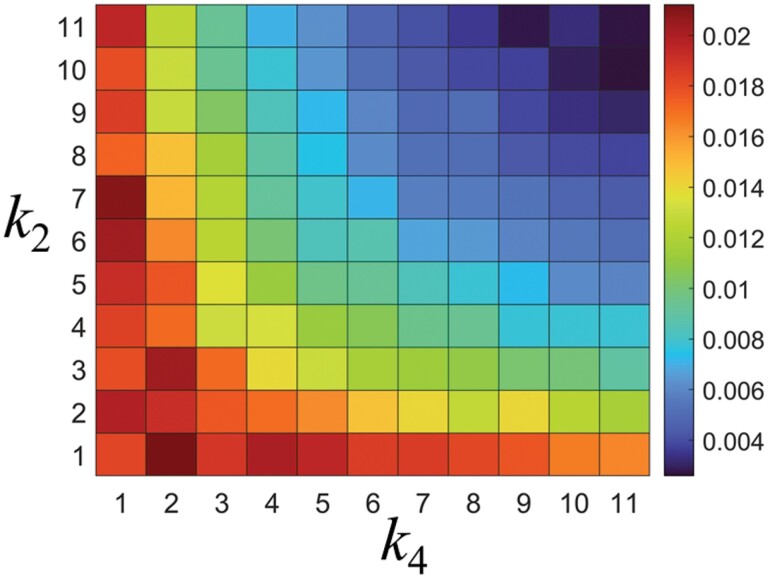
Food transport efficiency, *E*, for *k*_4_ and *k*_2_ where *k*_4_ and *k*_2_ = 1, 2, 3… 11.

Figure 11 shows the effect of *k*_4_ and *k*_2_ on *E*. For *k*_4_ > 1, *E* decreased sharply while increasing *k*_4_, and it also decreased sharply while increasing *k*_2_. Therefore, when *k*_4_ and *k*_2_ were increased together, *E* decreased in the direction of being minimized. This implies that both *k*_4_ and *k*_2_ have a dominant effect on *E*.

**Fig. 12.**
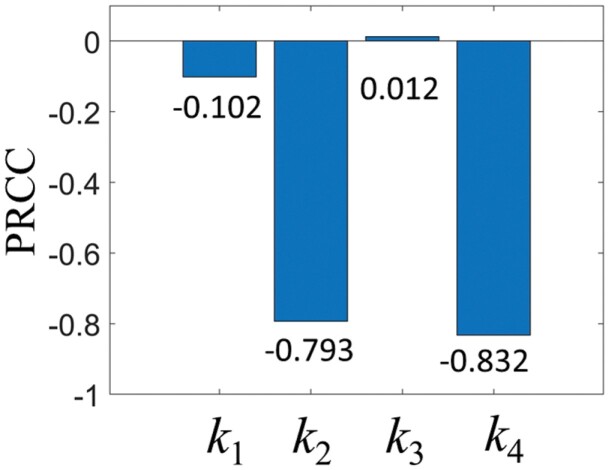
Partial rank correlation coefficient (PRCC) sensitivity analysis for *k*_1_, *k*_2_, *k*_3_, and *k*_4_.

As above, we investigated the effect of *k*_1_, *k*_2_, *k*_3_, and *k*_4_ on *E* for each variable combination. To quantitatively compare the effects, we performed a PRCC analysis (Fig. 12). *k*_1_, *k*_2_, and *k*_4_ had a negative effect on *E* while *k*_3_ had a positive effect. *k*_4_ had the biggest impact, followed by *k*_2_. In other words, *k*_4_ and *k*_2_ played almost decisive roles in the *E* value. *k*_1_ had the third largest effect, and *k*_3_ showed the relatively smallest effect.

## Discussion

Subterranean termites build tunnels below the ground for foraging. From an evolutionary perspective, it is believed that termites have evolved their behavior in a way that maximizes foraging efficiency, which includes both food search and transport efficiency. Otherwise, it would be difficult for the termites to maintain the stability of their tunnel system, given that the tunnel sizes often exceed 100 meters.

To study foraging efficiency, researchers have focused on both food search and transport efficiency. While food search efficiency has been actively studied due to its practical importance in termite pest management, transport efficiency has received less attention because of the difficulties in observing transport behavior.

However, some experimental studies of food search efficiency have provided a basic understanding of transport efficiency. These studies mainly focused on the relationship between soil environmental conditions, tunnel patterns, and transport behavior, which are directly related to transport efficiency (Evans 2003, Arab and Costa-Leonardo 2005, Bardunias and Su 2010, Lima and Costa-Leonardo 2012, Lee et al. 2020, Mizumoto et al. 2020).

In this study, we developed a termite individual-based model to explore the transport efficiency based on the insights provided by previous experimental findings and the aim to overcome the observational challenges. In this model, we defined food transport efficiency as the amount of food transported by termites from the foraging site to the nest within a specified period of time. However, it is known that termites share food with other members of the colony through the trophalaxis (Suárez and Thorne 2000, Huang et al. 2008, Du et al. 2016, Lee et al. 2021). Thus, termites may employ multiple strategies. Since a more in-depth investigation of this has not been conducted experimentally, the transport process studied in this model should be understood in a limited context. In this regard, it may be more accurate to view the nest site as a temporary transfer point for food during the trophalaxis process, rather than a storage location for all acquired food. In our model, the trophalaxis process was incorporated in Section *Food Transport Behavior of Simulated Termites* as the reduction of food during the transfer of food between two individuals.

The model was characterized by interactions between individuals and interactions between individuals and tunnels. The individual-tunnel interaction was influenced by four control variables: tunnel curvature, the density and distribution of tunnel surface irregularities, and the time termites spend stopping at irregularity sites.

The simulation results indicated that as the density of irregularities increased, the frequency of stopping behavior increased, leading to a decrease in transport efficiency. The increase in stopping behavior also led to an increase in the frequency of traffic jams, further reducing efficiency (as shown in Figs. 7 and 10). In some field conditions, the density of irregularities can be very high, causing transport efficiency to be very low. From an evolutionary perspective, it is likely that termites have evolved mechanisms to prevent this decrease in efficiency. Figure 10 illustrates one such mechanism. This figure shows that the frequency of jamming initially increases as the density of irregularities increases, but then decreases. Interestingly, this reduction in jamming frequency leads to a decrease in the number of encounters between simulated termites, serving as a brake to mitigate the decline in transport efficiency.

Figure 12 demonstrates that the distribution of irregularities has a negligible effect on transport efficiency. This may serve as a mechanism to maintain the robustness of transport efficiency in the face of varying irregularity distributions that can occur in soils with high heterogeneity. In addition, Lee et al. (2008) reported that when the size of a square-shaped irregularity is less than 2 mm in depth and less than 5 mm in width, termites are able to pass through it without stopping. This provides evidence for a mechanism that prevents transport efficiency from becoming too low in the presence of high soil heterogeneity.

Through simulations, Lee et al. (2018) showed that higher soil heterogeneity forms more branching tunnels, which increase the food finding probability by covering unexplored spaces between primary tunnels. At the same time, however, the same soil heterogeneity decreases transport efficiency. The relationship between termite ecosystem stability and termite foraging strategies may be better understood by exploring the optimization problem between these two efficiencies.

The model in this study assumes that termite tunnels have a sinusoidal shape, which is likely to be valid for homogenous soil conditions and short tunnel sections. To make the results of this study more realistic and meaningful, it is necessary to investigate simulation results in a variety of tunnel types. The model includes a simulation rule that a traffic jam occurs when 4 simulated termites overlap on the same site and the sum of their direction vectors is 0. This number of simulated termites (4) was determined from observed traffic jamming in a straight tunnel with a length of 10 cm and a width of 3 mm (Fig. 5). However, actual termite tunnels vary in width and length (Campora and Grace 2004, Bardunias and Su 2010), so this rule may not always be accurate. The model could be improved in the future with more experimental data on traffic jamming.

It is important to note that this model is limited in its inclusion of termite-specific behavior and various environmental conditions. Nevertheless, this study is valuable in that it explores food transport efficiency, which has been rarely studied, and provides insight into research optimizing foraging efficiency.
